# An autoantibody signature targeting cuproptosis-related proteins for non-small cell lung cancer detection and prognosis

**DOI:** 10.7717/peerj.21260

**Published:** 2026-05-27

**Authors:** Aichen Liu, Lulu Zhang, Peiqi Yu, Tingzun Nie, Xiaobin Cao, Jing Li, Wenke Sun, Yihao Liang, Songyun Ouyang, Liping Dai, Jingjing Liu

**Affiliations:** 1 Henan Institute of Medical and Pharmaceutical Sciences, Zhengzhou University, Zhengzhou City, Henan Province, China; 2 Henan Key Medical Laboratory of Tumor Molecular Biomarkers, Zhengzhou University, Zhengzhou City, Henan Province, China; 3 First Affiliated Hospital of Zhengzhou University, Zhengzhou City, Henan Province, China

**Keywords:** Autoantibody, Cuproptosis-related proteins, Non-small cell lung cancer, Detection and prognosis

## Abstract

**Background:**

Autoantibodies against tumor-associated antigens in plasma are valuable biomarkers for early cancer detection and prognostic stratification. Dihydrolipoamide acetyltransferase (DLAT) and lipoic acid synthetase (LIAS), two key cuproptosis regulators, are abnormally expressed in non-small cell lung cancer (NSCLC) and are potential biomarkers for clinical diagnosis. This study explored the significance of anti-DLAT and anti-LIAS autoantibodies in the clinical diagnosis and prognosis of NSCLC.

**Methods:**

Plasma levels of anti-DLAT and anti-LIAS autoantibodies were detected using Enzyme-Linked Immunosorbent Assay (ELISA). Their diagnostic value was evaluated in 340 cases with normal control (NC), 260 patients with benign pulmonary nodule (BPN) and 340 patients with NSCLC. Additionally, the prognostic value of these autoantibodies was analyzed in a separate independent cohort of 354 patients with NSCLC.

**Results:**

The expression levels of anti-DLAT and anti-LIAS autoantibodies were significantly elevated in NSCLC compared to those in BPN and NC. These autoantibodies distinguished NSCLC from NC with AUCs of 0.712 (95% CI [0.669–0.756]) and 0.668 (95% CI [0.623–0.714]), respectively. To enhance diagnostic efficacy, a multi-autoantibody signature (anti-DLAT/LIAS/FDX1/COPT1) was constructed, which significantly improved discrimination (NSCLC *vs* NC: AUC = 0.805; NSCLC *vs* BPN: AUC = 0.751). Prognostic analysis indicated that anti-LIAS autoantibody served as an independent predictor of outcome (HR = 1.42, 95% CI [1.01–1.99]).

**Conclusions:**

These findings demonstrate the clinical utility of an autoantibody signature targeting cuproptosis-related proteins for NSCLC diagnosis and prognosis.

## Introduction

Lung cancer remains the most prevalent and lethal malignancy worldwide, accounting for the highest cancer-related mortality rate among all cancer types (Bray et al., 2024). Non-small cell lung cancer (NSCLC) is the most common type of lung cancer, accounting for about 85% of all lung cancer cases (Arabnejad, Tothill & Chianella, 2024; Bray et al., 2024). The stark prognostic disparity between early-stage and advanced NSCLC is exemplified by the 83% *versus* 14% five-year survival rates, respectively. This disparity highlights the urgent need for enhanced early detection strategies. Emerging research has increasingly focused on tumor-associated autoantibodies (TAAbs) as promising immunological markers of NSCLC (Cai et al., 2022a).

Serum autoantibodies are immunoglobulins produced against self tissues or cellular components (Zeng et al., 2024). Normally, the immune system recognizes and attacks foreign substances (such as pathogens) to protect the body from infection (Wang et al., 2024). However, during malignant transformation, the recognition mechanism of the immune system becomes dysregulated, mistakenly attacking its own tissues or cellular components as “foreign substances,” thereby producing antibodies against self, which are called autoantibodies (Zeng et al., 2024). Autoantibodies hold promise as biomarkers due to their stability and prolonged detectability. Clinical studies have identified distinct autoantibody signatures in the sera of NSCLC patients, highlighting their potential clinical utility for early detection, prognostic stratification, therapeutic response monitoring, and immunotherapy guidance (Wang et al., 2024).

Copper (Cu), an essential trace element in the human body (Xie et al., 2024), modulates tumor-associated signaling pathways and biological behaviors (Liu et al., 2024b). Tsvetkov et al. (2022) defined a new type of copper-dependent cell death in 2022, termed cuproptosis. This mode of cell death is induced by mitochondrial copper overload, which in turn causes the accumulation of acylated dihydrolipoyl transacetylase (DLAT), an acyltransferase of the tricarboxylic acid (TCA) cycle. Through whole-genome CRISPR knockout screening, 13 genes are referred to as cuproptosis key genes, including CDKN2A, FDX1, DLD, DLAT, LIAS, GLS, LIPT1, MTF1, PDHA1, PDHB, ATP7A, ATP7B, and SLC31A1 (Tsvetkov et al., 2022). Lipoic acid synthetase (LIAS) provides essential lipoylation modifications for DLAT, determining copper toxicity effects that culminate in cell death (Huo et al., 2023). LIAS facilitates the synthesis of mitochondrial metabolic enzymes that are essential for energy metabolism and antioxidant responses (Liao et al., 2024), while DLAT catalyzes pyruvate-to-acetyl-CoA conversion in the TCA cycle (Zhao & Qi, 2023). Thus, both proteins are central to cellular bioenergetics and redox regulation (Xu et al., 2023). The expression of DLAT and LIAS significantly predicts the metastasis and prognosis of NSCLC (Cai et al., 2022b; Ma et al., 2024). Tumor-Associated Antigens (TAAs) exhibit significant limitations as biomarkers, including low expression in plasma and short circulation time (Zhu et al., 2013). In contrast, autoantibodies against TAAs have the advantages of high stability, specificity and sensitivity (Belousov, 2022). However, no relevant studies have investigated whether autoantibody responses target DLAT and LIAS in patients with NSCLC. Consequently, the clinical utility of the anti-DLAT and anti-LIAS autoantibodies in NSCLC remains unclear.

In this study, we systematically investigated the diagnostic and prognostic potential of autoantibodies against key cuproptosis-related proteins in NSCLC. We initially characterized the expression profiles and potential diagnostic value of the anti-DLAT and anti-LIAS autoantibodies. To optimize the diagnostic performance, we integrated these findings with our previously identified cuproptosis-related biomarkers (anti-FDX1 and anti-COPT1 autoantibodies) (Cao et al., 2025; Li et al., 2025) to construct an autoantibody signature. Finally, the prognostic relevance of this autoantibody signature, which incorporated all four biomarkers (anti-DLAT, anti-LIAS, anti-FDX1, and anti-COPT1 autoantibodies) was evaluated in the prognostic cohort.

## Materials & Methods

### Study design

The study design is illustrated in Fig. S1. The plasma samples were divided into the verification, validation, and prognostic groups. First, the expression levels of anti-DLAT and anti-LIAS autoantibodies were detected using enzyme-linked immunosorbent assay (ELISA) in the verification and validation groups. Subsequently, the diagnostic efficacy of anti-DLAT and anti-LIAS autoantibodies in distinguishing NSCLC from controls was analyzed. In our previous studies, we evaluated the expression levels of anti-FDX1 and anti-COPT1 autoantibodies in a cohort that partially overlapped the validation set used in the current study (Cao et al., 2025; Li et al., 2025). We constructed an autoantibody signature using anti-DLAT, anti-LIAS, anti-FDX1, and anti-COPT1 autoantibodies for NSCLC detection in the validation group. Finally, the expression levels of anti-DLAT, anti-LIAS, anti-FDX1, and anti-COPT1 autoantibodies were detected by ELISA in a prognostic group of 354 patients with NSCLC. We evaluated the prognostic value of the autoantibody signature using univariate and multivariate Cox regression analyses, and constructed a prognostic model.

### Sample collection

The samples were collected from the First Affiliated Hospital of Zhengzhou University (Zhengzhou, China). The sample cohort consisted of patients with NSCLC, patients with benign pulmonary nodule (BPN) and normal control (NC). There were 80 NSCLC and 80 NC cases in the verification group, and 260 NSCLC, 260 BPN, and 260 NC cases in the validation group. The demographic information of the participants is presented in Table 1. Finally, the prognostic analysis was performed on 354 patients with NSCLC in the prognostic group (Table S1). All samples were obtained from individuals over 18 years of age, and no immune-mediated diseases were identified. The study was approved by the Ethics Committee of the First Affiliated Hospital of Zhengzhou University (2021-KY-1057-002), and all participants signed informed consent forms. Detailed information on the clinical characteristics of the patients was also collected.

**Table 1:** Clinical characteristics of the verification and validation group.

	Verification group (*N* = 160)			Validation group (*N* = 780)			
Variables	NSCLC (*n* = 80)	NC (*n* = 80)	*P*	NSCLC (*n* = 260)	BPN (*n* = 260)	NC (*n* = 260)	*P*
**Age (year), n (%)**							
<50y	15 (18.75)	21 (26.25)	>0.05	62 (23.85)	86 (33.08)	59 (22.69)	>0.05
50–80y	63 (78.75)	58 (72.50)		196 (75.38)	169 (65.00)	192 (73.85)	
>80y	2 (2.50)	1 (1.25)		2 (0.77)	5 (1.92)	9 (3.46)	
Mean ± SD	55 ± 11	60 ± 11		56 ± 11	55 ± 12	55 ± 11	
**Gender, n (%)**							
Male	41 (51.25)	41 (51.25)	>0.05	158 (60.77)	158 (60.77)	158 (60.77)	>0.05
Female	39 (48.75)	39 (48.75)		102 (39.23)	102 (39.23)	102 (39.23)	
**Histology, n (%)**							
LUAD	59 (73.75)			191 (73.46)			
LUSC	9 (11.25)			42 (16.15)			
Other NSCLC	12 (15.00)			27 (10.38)			
Fibrotic nodules					195 (75.00)		
Granulomas					22 (8.46)		
Others					43 (16.54)		
**Smoking, n (%)**							
Yes	23 (28.75)			87 (33.46)	67 (25.77)		
No	54 (67.50)			172 (66.15)	186 (71.54)		
Unknown	3 (3.75)	80 (100)		1 (0.39)	7 (2.69)	260 (100)	
**Drinking, n (%)**							
Yes	11 (13.75)			65 (25.00)	56 (21.54)		
No	66 (82.50)			191 (73.46)	197 (75.77)		
Unknown	3 (3.75)	80 (100)		4 (1.54)	7 (2.69)	260 (100)	
**Lymph node metastasis, n (%)**							
Yes	43 (53.75)			77 (29.62)			
No	37 (46.25)			162 (62.31)			
Unknown	0 (0)			21 (8.08)			
**Distant metastasis, n (%)**							
Yes	43 (53.75)			31 (11.92)			
No	30 (37.50)			198 (76.15)			
Unknown	7 (8.75)			31 (11.92)			
**Clinical stage, n (%)**							
I	27 (33.75)			137 (52.69)			
II	10 (12.50)			21 (8.08)			
III	0 (0)			34 (13.08)			
IV	43 (53.75)			30 (11.54)			
Unknown	0 (0)			38 (14.62)			
**Tumor/lesion size** ** (median and range), (mm)**				20 (4–98)	16 (2–85)		

**Notes.**

NSCLC non-small cell lung cancer BPN benign pulmonary nodule NC normal control SD standard deviation

Smoking and drinking history was not provided for the NC group, and these variables are therefore listed as “Unknown” for all NC participants.

### Sample processing

All blood samples were collected in five mL vacuum blood collection tubes (containing EDTA-K2 anticoagulant). The subjects, in a fasting state, had 3–4 mL of venous blood drawn from the antecubital elbow vein and the tubes were inverted 5–8 times to ensure thorough mixing of the blood and anticoagulant. The samples were then centrifuged at 1,000 × g for 5 min at room temperature, and the supernatant plasma was aspirated with a disposable pipette and aliquoted into 1.5 mL Eppendorf tubes. After detailed labeling of the disease type and number, sampling date, and sample source, the plasma samples were immediately stored in a −80 °C ultra-low temperature freezer. When needed, the required samples were thawed in advance in a 4 °C refrigerator to avoid repeated freeze-thaw cycles in the original tube.

### Enzyme-Linked Immunosorbent Assay

Recombinant DLAT, LIAS, and COPT1 proteins were purchased from Cloud-Clone Corp (RPA696Hu01, RPG355Hu01, and RPE493Hu01, WuHan, China). Recombinant FDX1 protein was purchased from CUSABIO (CSB-EP008570HU, WuHan, China). We detected the expression levels of the four autoantibodies using Enzyme-Linked Immunosorbent Assay (ELISA). The specific method was as follows: recombinant proteins were coated onto 96-well microtiter plates at a concentration of 0.125 µg/mL as antigens, plasma samples were diluted at a ratio of 1:100 as the primary antibody, and the secondary antibody was horseradish peroxidase (HRP)-labeled goat anti-human IgG, which was diluted at a ratio of 1:10,000. Plasma samples were assayed in single wells. Each plate contained two duplicate quality-control wells and two blank controls to ensure the stability and accuracy of all well OD values. Specific binding index (SBI) values were used to determine the expression levels of the autoantibodies in different groups. SBI = (OD value of sample to be tested—mean OD value of the blank wells)/(mean OD value of the quality control samples—mean OD value of the blank wells). Blood samples from 100 normal controls were pooled for quality control, which was used to minimize inter-plate variation and represent the general population.

### Statistical analysis

SPSS 21.0 (IBM Corp., Armonk, NY, USA), RStudio and GraphPad Prism (version 8.0) were used for statistical analysis and visualization of the experimental results. *T*-tests were used for age and sex matching, Mann–Whitney *U* tests were used to compare the expression levels of anti-DLAT and anti-LIAS autoantibodies in NSCLC and different clinical features of NSCLC, and chi-square tests were used to compare the positivity rates between the two groups. Receiver operating characteristic (ROC) curve analysis was performed to evaluate the differential diagnostic values, area under the curve (AUC), 95% confidence interval (95% CI), sensitivity, specificity, and Youden index (YI). Logistic regression analysis was used to construct the combined models. The prognostic value of anti-DLAT, anti-LIAS, anti-FDX1, and anti-COPT1 autoantibodies for NSCLC was evaluated using Kaplan–Meier survival curve analysis supplemented by Cox regression analysis with hazard ratios (HR). All results were considered statistically significant at *P* < 0.05 and AUC > 0.5. The SBI value corresponding to the maximum Youden index was selected as the cut-off value for statistical analysis.

## Results

### The expression levels of anti-DLAT and anti-LIAS autoantibodies were detected in the verification group

**Figure 1:**
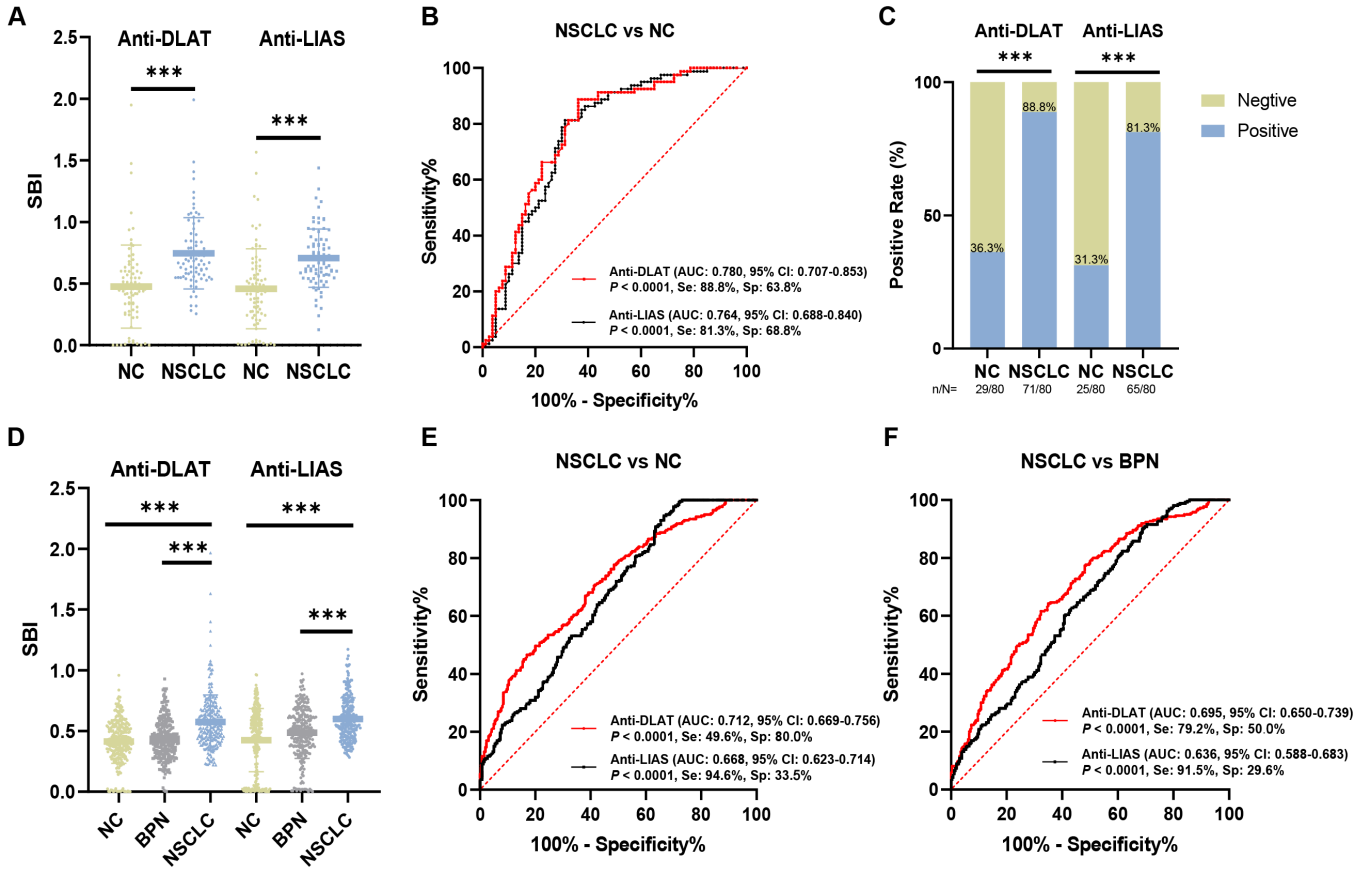
The expression levels of anti-DLAT and anti-LIAS autoantibodies were confirmed in the verification and validation group. (A) The expression levels of anti-DLAT and anti-LIAS autoantibodies in the verification group. (B) ROC curves of anti-DLAT and anti-LIAS autoantibodies in the verification group. (C) Positive rates of anti-DLAT and anti-LIAS autoantibodies in the verification group. (D) The expression levels of anti-DLAT and anti-LIAS autoantibodies in the validation group. (E–F) ROC curves analysis of anti-DLAT and anti-LIAS autoantibodies for distinguishing NSCLC from NC and BPN. Se, sensitivity; Sp, specificity; ^∗∗∗^, *P* < 0.001.

Indirect ELISA was performed to detect the presence of autoantibodies against DLAT and LIAS in NSCLC and NC plasma samples. The demographic information of the participants is presented in Table 1. In the verification group, the expression levels of anti-DLAT and anti-LIAS autoantibodies were significantly higher in patients with NSCLC than in NC patients (Fig. 1A). The AUC values for anti-DLAT and anti-LIAS autoantibodies were 0.780 (95% CI [0.707–0.853], sensitivity = 88.8%, specificity = 63.8%) and 0.764 (95% CI [0.688–0.840], sensitivity = 81.3%, specificity = 68.8%), respectively (Fig. 1B). In the verification group, the positivity rate was calculated using the maximum Youden index as the cut-off value (anti-DLAT autoantibody: cut-off value = 0.516; anti-LIAS autoantibody: cut-off value = 0.540). The positive frequency of anti-DLAT autoantibody in NSCLC was 88.8% (71/80), whereas it was only 36.3% (29/80) in NC. The positive frequency of anti-LIAS autoantibody in NSCLC was 81.3% (65/80) and 31.3% (25/80) in NC, with a statistically significant difference (Fig. 1C).

### The expression levels of anti-DLAT and anti-LIAS autoantibodies were confirmed in the validation group, which can distinguish NSCLC from NC and BPN

To further validate these results, the expression levels of anti-DLAT and anti-LIAS autoantibodies were measured in the validation group consisting of 260 patients with NSCLC, 260 patients with BPN, and 260 NC individuals (Table 1). The expression levels of anti-DLAT and anti-LIAS autoantibodies were higher in patients with NSCLC (Fig. 1D). Anti-DLAT and anti-LIAS autoantibodies significantly distinguished NSCLC patients from NC patients, with AUCs of 0.712 (95% CI [0.669–0.756], sensitivity = 49.6%, specificity = 80.0%) and 0.668 (95% CI [0.623–0.714], sensitivity = 94.6%, specificity = 33.5%), respectively (Fig. 1E). Furthermore, anti-DLAT and anti-LIAS autoantibodies could differentiate NSCLC from BPN with AUCs of 0.695 (95% CI [0.650–0.739], sensitivity = 79.2%, specificity = 50.0%) and 0.636 (95% CI [0.588–0.683], sensitivity = 91.5%, specificity = 29.6%), respectively (Fig. 1F). These findings indicate the diagnostic values of anti-DLAT and anti-LIAS autoantibodies in effectively distinguishing NSCLC from NC and BPN.

### Anti-DLAT and anti-LIAS autoantibodies can distinguish NSCLC with clinical subtypes from NC

**Figure 2:**
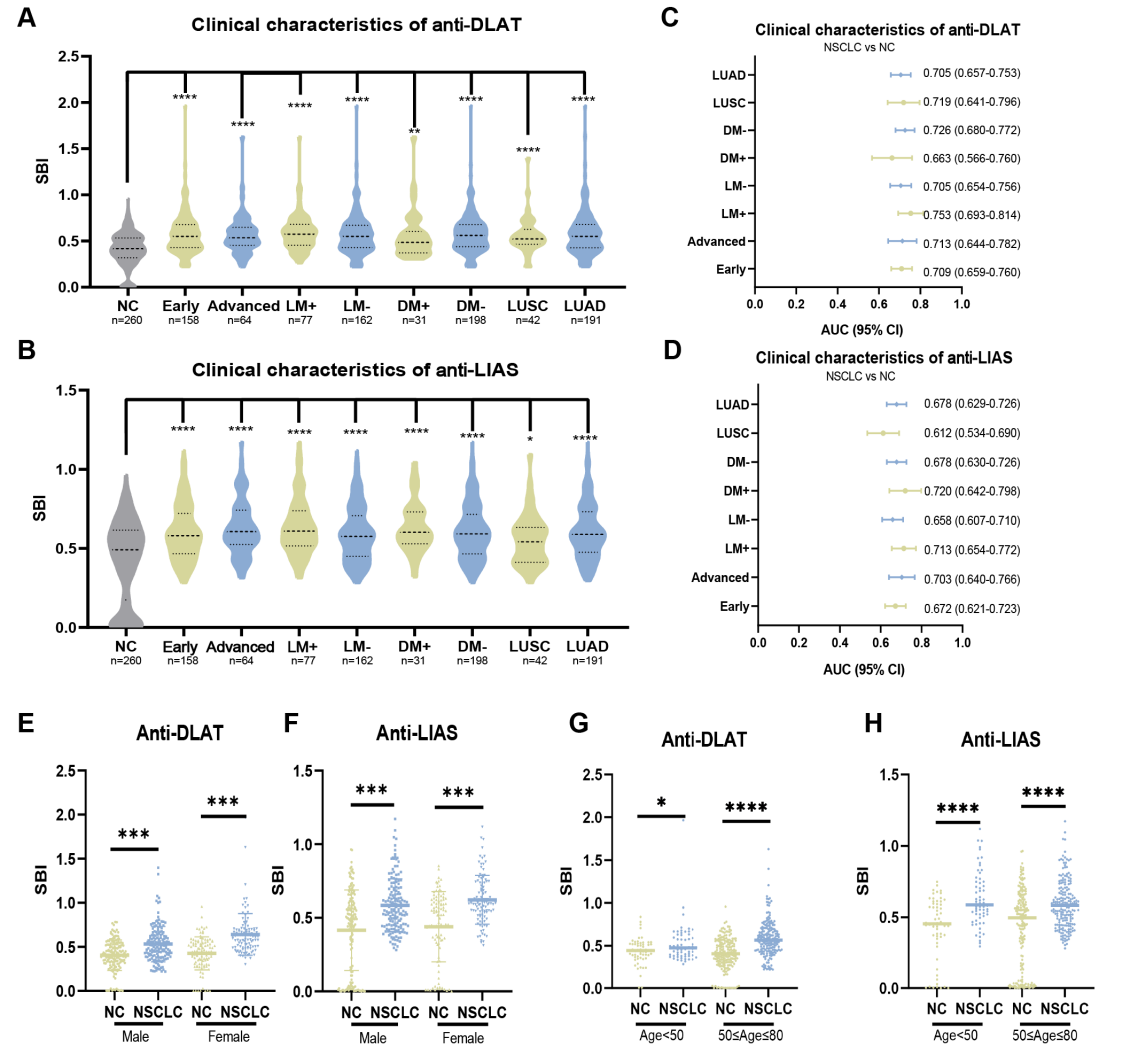
Anti-DLAT and anti-LIAS autoantibodies can distinguish NSCLC with clinical subtypes from NC. (A, B) The expression levels of anti-DLAT and anti-LIAS autoantibodies in different clinical subgroups. (C, D) AUC and 95% CI of anti-DLAT and anti-LIAS autoantibodies in different clinical subgroups, used to distinguish different clinical characteristics and NC. (E–H) The expression levels of anti-DLAT and anti-LIAS autoantibodies stratified by age and gender. Early, patients with early NSCLC (clinical stage I & II); Advanced, patients with advanced NSCLC (clinical stage III & IV); LM+, lymph node metastasis positive; LM-, lymph node metastasis negative; DM+, distant metastasis positive; DM-, distant metastasis negative; LUAD, lung adenocarcinoma; LUSC, lung squamous cell carcinoma. ^∗^, *P* < 0.05; ^∗∗^, *P* < 0.01; ^∗∗∗^, *P* < 0.001 , ^∗∗∗∗^, *P* < 0.0001.

We divided 260 patients with NSCLC in the validation group into different clinical subgroups based on cancer stage, lymph node metastasis (LM), distant metastasis (DM), and histological type. In every subgroup, both anti-DLAT and anti-LIAS autoantibodies expression levels were higher in NSCLC (*P* < 0.05) (Figs. 2A–2B). Within the different subgroups, there were no significant differences in the expression levels of anti-DLAT and anti-LIAS autoantibodies (*P* > 0.05) (Fig. S2). The AUC range for anti-DLAT autoantibody in various clinical subgroups was 0.663–0.753 (Fig. 2C, Fig. S3). For anti-LIAS autoantibody, the AUC range in different clinical subgroups was 0.612–0.720 (Fig. 2D, Fig. S3). Anti-DLAT and anti-LIAS autoantibodies could distinguish patients with early-stage NSCLC from NC, with AUC values of 0.709 (95% CI [0.659–0.760], sensitivity = 46.8%, specificity = 83.1%) and 0.672 (95% CI [0.621–0.723], sensitivity = 94.9%, specificity = 33.5%), respectively (Figs. 2C–2D). These results identify anti-DLAT and anti-LIAS autoantibodies as potential biomarkers for early-stage NSCLC.

Pairwise comparisons of different subgroups stratified by age and sex status were performed. In the different subgroups, anti-DLAT and anti-LIAS autoantibodies expression levels were higher in NSCLC than in NC (Figs. 2E–2H). The AUC values of anti-DLAT and anti-LIAS autoantibodies to distinguish female NSCLC from NC were 0.783 (95% CI [0.721–0.844], sensitivity = 59.8%, specificity = 82.4%) and 0.701 (95% CI [0.630–0.771], sensitivity = 85.3%, specificity = 47.1%), respectively, which were higher than those for males (Figs. S4A–S4D). Age stratification (50≤age≤80 years) showed enhanced anti-DLAT autoantibody diagnostic capacity (AUC = 0.743, 95% CI [0.695–0.792], sensitivity = 81.1%, specificity = 56.3%) compared to younger individuals (Figs. S4E–S4F), whereas anti-LIAS autoantibody exhibited reduced efficacy (AUC = 0.651, 95% CI [0.596–0.705], sensitivity = 98.0%, specificity = 32.3%) relative to younger groups (Figs. S4G– S4H). The diagnostic values of anti-DLAT and anti-LIAS autoantibodies are affected by age and sex.

### Anti-DLAT and anti-LIAS autoantibodies can distinguish NSCLC with clinical subtypes from BPN

**Figure 3:**
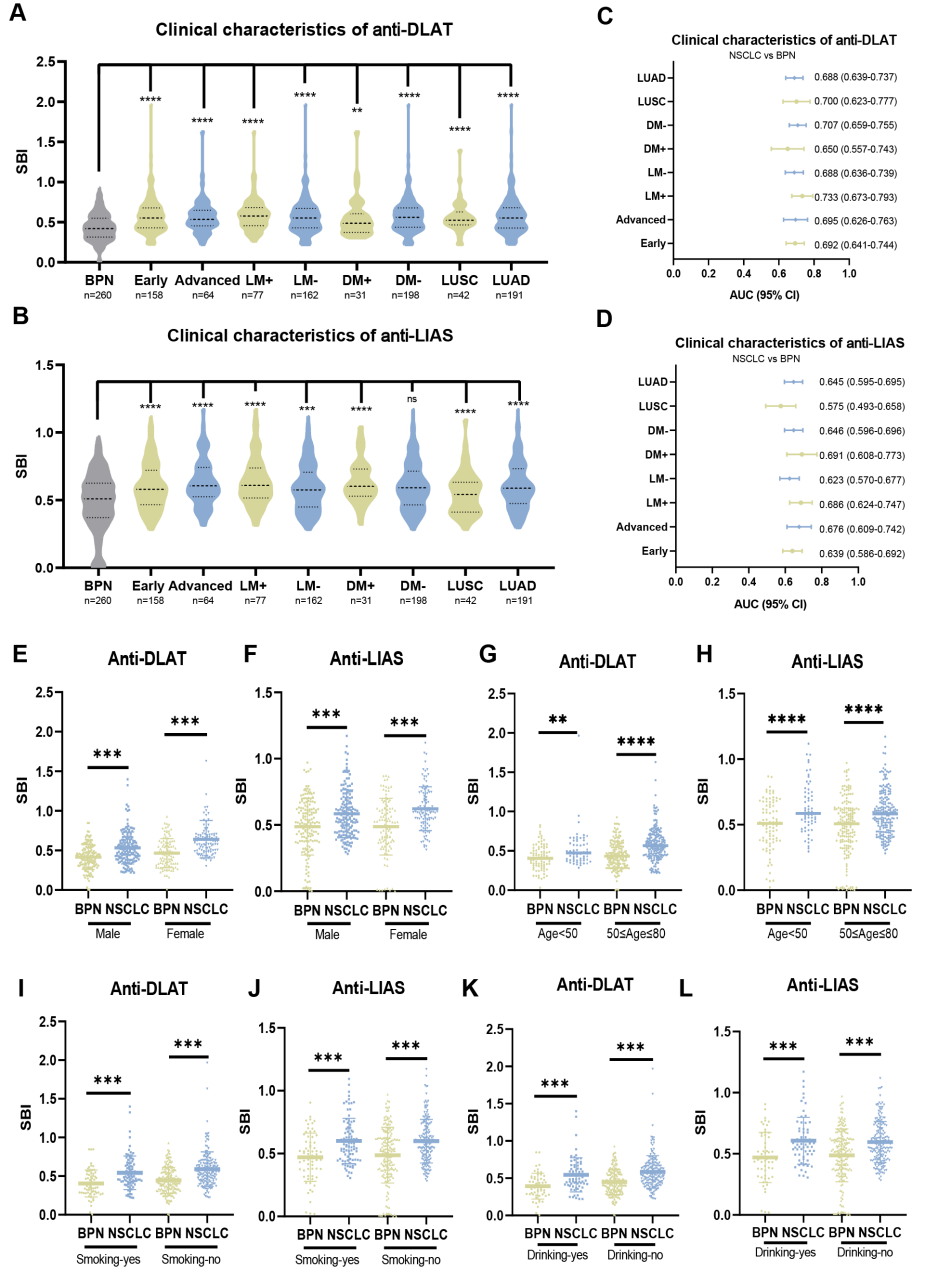
Anti-DLAT and anti-LIAS autoantibodies can distinguish NSCLC with clinical subtypes from BPN. (A, B) The expression levels of anti-DLAT and anti-LIAS autoantibodies in different clinical subgroups. (C, D) AUC and 95% CI of anti-DLAT and anti-LIAS autoantibodies in different clinical subgroups, used to distinguish different clinical characteristics and BPN. (E–L) The expression levels anti-DLAT and anti-LIAS autoantibodies stratified by age, gender, smoking and drinking status. Early, patients with early NSCLC (clinical stage I & II); Advanced, patients with advanced NSCLC (clinical stage III & IV); LM+, lymph node metastasis positive; LM-, lymph node metastasis negative; DM+, distant metastasis positive; DM-, distant metastasis negative; Smoking yes/no, individuals with or without smoking history; Drinking yes/no, individuals with or without drinking history. ^∗∗^, *P* < 0.01; ^∗∗∗^, *P* < 0.001, ^∗∗∗∗^, *P* < 0.0001.

Compared to the BPN group, NSCLC patients with different clinical characteristics had significantly higher expression levels of anti-DLAT and anti-LIAS autoantibodies (Figs. 3A–3B). The AUC values of anti-DLAT autoantibody in NSCLC with different clinical features ranged from 0.650 to 0.733, whereas the AUC values of anti-LIAS autoantibody ranged from 0.575 to 0.691 (Figs. 3C–3D, Fig. S5). Anti-DLAT and anti-LIAS autoantibodies could distinguish early-stage NSCLC patients from patients with BPN, with AUC values of 0.692 (95% CI [0.641–0.744], sensitivity = 78.5%, specificity = 50.0%) and 0.639 (95% CI [0.586–0.692], sensitivity = 90.8%, specificity = 30.4%), respectively (Figs. 3C–3D). These results indicate the potential of anti-DLAT and anti-LIAS autoantibodies in discriminating early-stage NSCLC from BPN.

Pairwise comparisons of the different subgroups according to age, sex, smoking status, and drinking status were performed. In the different subgroups, the expression levels of anti-DLAT and anti-LIAS autoantibodies in patients with NSCLC were significantly higher than those in patients with BPN (Figs. 3E–3L). The AUC values of anti-DLAT and anti-LIAS autoantibodies for distinguishing female NSCLC from BPN were 0.735 (95% CI [0.667–0.804], sensitivity = 92.2%, specificity = 47.1%) and 0.676 (95% CI [0.603–0.750], sensitivity = 71.6%, specificity = 59.8%), respectively, which were higher than those for males (Figs. S6A–S6D). For individuals aged 50–80 years, the AUC value of the anti-DLAT autoantibody was 0.699 (95% CI [0.645–0.753], sensitivity = 66.8%, specificity = 65.1%), which was higher than that for young individuals. However, anti-LIAS autoantibody exhibited reduced efficacy (AUC = 0.630, 95% CI [0.573–0.688], sensitivity = 90.8%, specificity = 31.4%) in individuals aged 50–80 years relative to the younger groups (Figs. S6E–S6H). The anti-DLAT autoantibody diagnostic capacity remained stable across smoking and drinking stratifications (Figs. S6I–S6L). The anti-LIAS autoantibody had a higher diagnostic value (AUC = 0.670, 95% CI [0.584–0.756], sensitivity = 63.2%, specificity = 65.7%) for distinguishing NSCLC without smoking history from BPN (Figs. S6M–S6N). Regarding drinking status, anti-LIAS autoantibody exhibited enhanced diagnostic capacity in drinkers (AUC = 0.685, 95% CI [0.589–0.782]; sensitivity = 67.7%, specificity = 69.6%) compared to non-drinkers (Figs. S6O–S6P).

**Figure 4:**
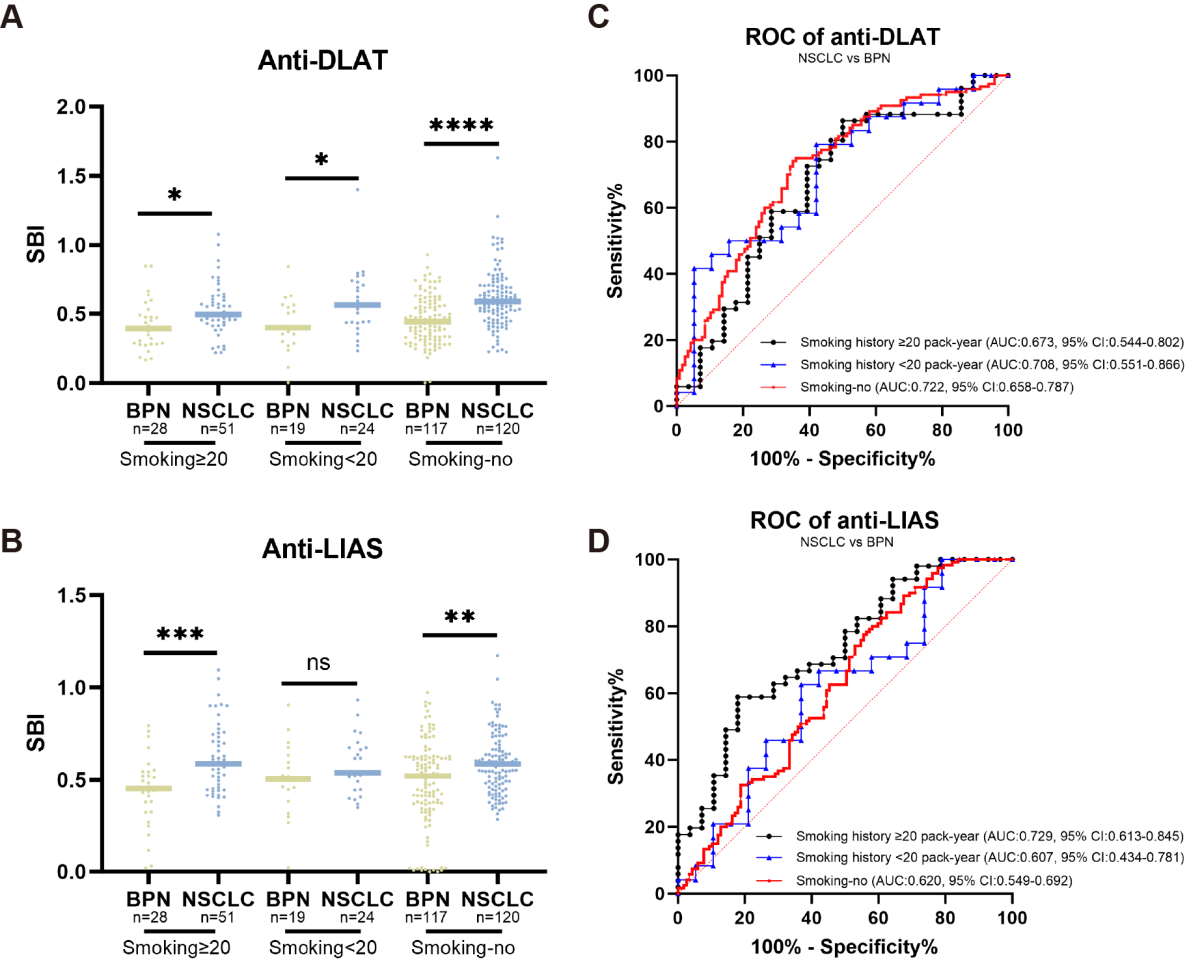
Anti-DLAT and anti-LIAS autoantibodies can distinguish NSCLC from BPN in individuals aged 50–80 years. (A–B) The expression levels of anti-DLAT and anti-LIAS autoantibodies in smoking individuals aged 50–80 years. (C) ROC curves of anti-DLAT autoantibody to distinguish NSCLC from BPN in smoking individuals aged 50–80 years. (D) ROC curves of anti-LIAS autoantibody to distinguish NSCLC from BPN in smoking individuals aged 50–80 years. Smoking ≥ 20, individuals with smoking history ≥ 20 pack-year; smoking <20, individuals with smoking history <20 pack-year; smoking no, individuals without smoking history. ns, not significant; *, *P* < 0.05; **, *P* < 0.01; ***, *P* < 0.001; ****, *P* < 0.0001.

Smoking is a critical factor influencing the development of lung cancer. Particularly, among individuals aged 50–80 years who have a 20 pack-year smoking history, the risk of lung cancer is significantly higher than that in the general population. Our study found that both anti-DLAT and anti-LIAS autoantibodies were highly expressed in patients with NSCLC from the high-risk smoking population (Figs. 4A–4B). Specifically, for individuals aged 50–80 years with a smoking history of ≥ 20 pack-year, the anti-DLAT autoantibody showed an AUC of 0.673 (95% CI [0.544–0.802], sensitivity = 86.3%, specificity = 50.0%) (Fig. 4C). The anti-LIAS autoantibody demonstrated an even higher diagnostic performance in distinguishing NSCLC from BPN in the same high-risk smoking population, with an AUC of 0.729 (95% CI [0.613–0.845], sensitivity = 58.8%, specificity = 82.1%) (Fig. 4D). Anti-DLAT and anti-LIAS autoantibodies can effectively distinguish benign from malignant in high-risk smokers.

### The combined diagnosis of the autoantibody signature incorporating anti-DLAT, anti-LIAS, anti-FDX1, and anti-COPT1 autoantibodies significantly improved the diagnostic efficiency

**Figure 5:**
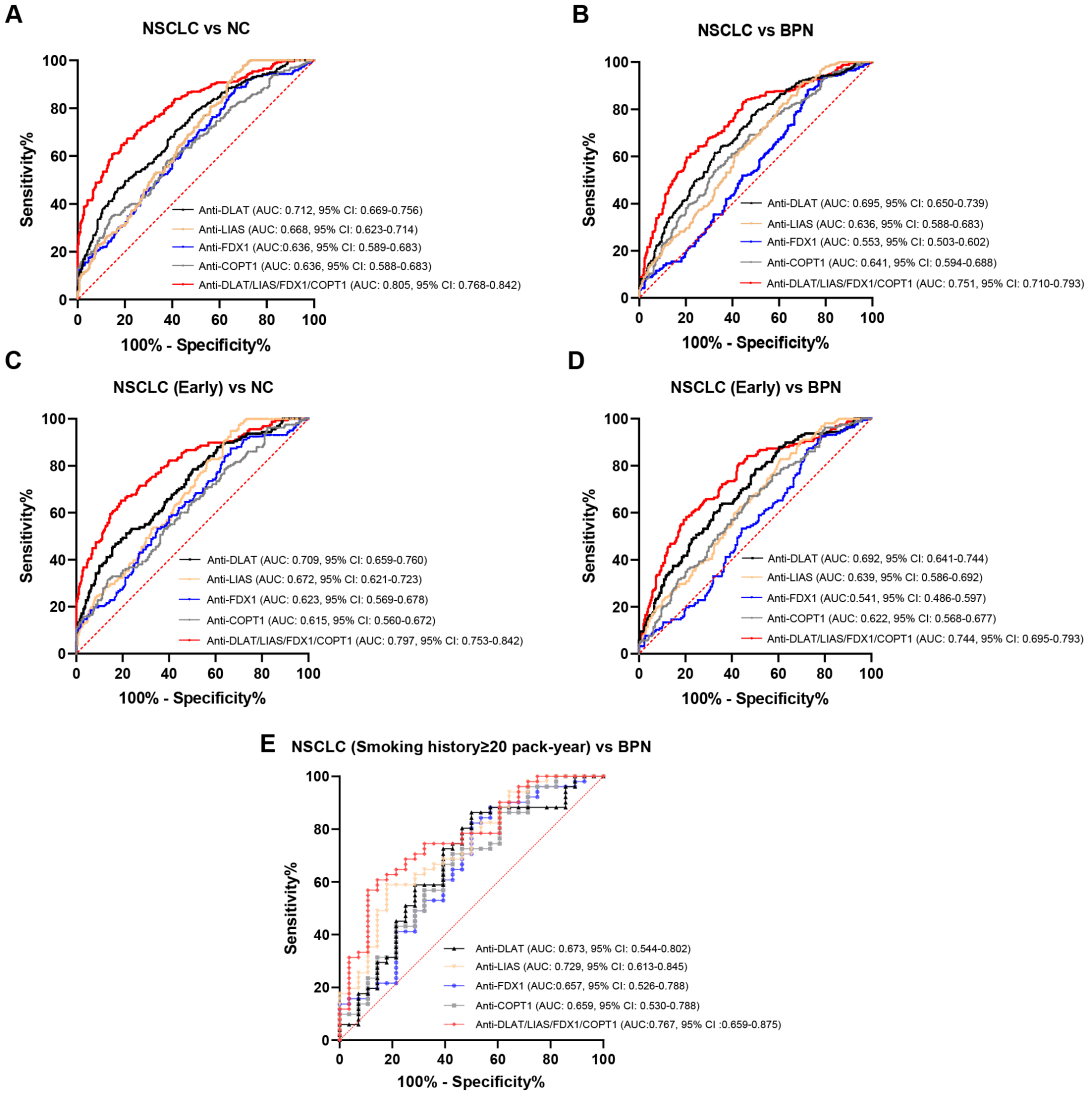
The diagnostic value was jointly evaluated by combining the autoantibody signature of anti-DLAT, anti-LIAS, anti-FDX1, and anti-COPT1 autoantibodies. (A) Combined diagnosis of four autoantibodies to distinguish NSCLC from NC. (B) Combined diagnosis of four autoantibodies to distinguish NSCLC from BPN. (C) Combined diagnosis of four autoantibodies to distinguish early NSCLC from NC. (D) Combined diagnosis of four autoantibodies to distinguish early NSCLC from BPN. (E) Combined diagnosis of four autoantibodies to distinguish NSCLC from BPN in a population with a ≥ 20 pack-year of smoking history.

We initially characterized the expression profiles and potential diagnostic value of the anti-DLAT and anti-LIAS autoantibodies. To optimize the diagnostic performance, we integrated these findings with our previously identified biomarkers (anti-FDX1 and anti-COPT1 autoantibodies) (Cao et al., 2025; Li et al., 2025) to construct an autoantibody signature. The AUC values of the autoantibody signature distinguishing NSCLC from NC and BPN increased to 0.805 (95% CI [0.768–0.842], sensitivity = 67.3%, specificity = 79.2%) and 0.751 (95% CI [0.710–0.793], sensitivity = 61.2%, specificity = 78.1%), respectively (Figs. 5A–5B, Table S2). Moreover, the AUC values of this autoantibody signature for distinguishing early-stage NSCLC patients from NC and BPN were 0.797 (95% CI [0.753–0.842], sensitivity = 65.2%, specificity = 80.8%) and 0.744 (95% CI [0.695–0.793], sensitivity = 81.0%, specificity = 56.9%), respectively (Figs. 5C–5D, Table S3). Furthermore, the autoantibody signature achieved an AUC of 0.767 (95% CI [0.659–0.875], sensitivity = 60.8%, specificity = 85.7%) in distinguishing NSCLC from BPN among individuals aged 50–80 years with a smoking history of ≥20 pack-year (Fig. 5E, Table S3).

### The impact of the autoantibody signature on the prognosis of NSCLC

**Figure 6:**
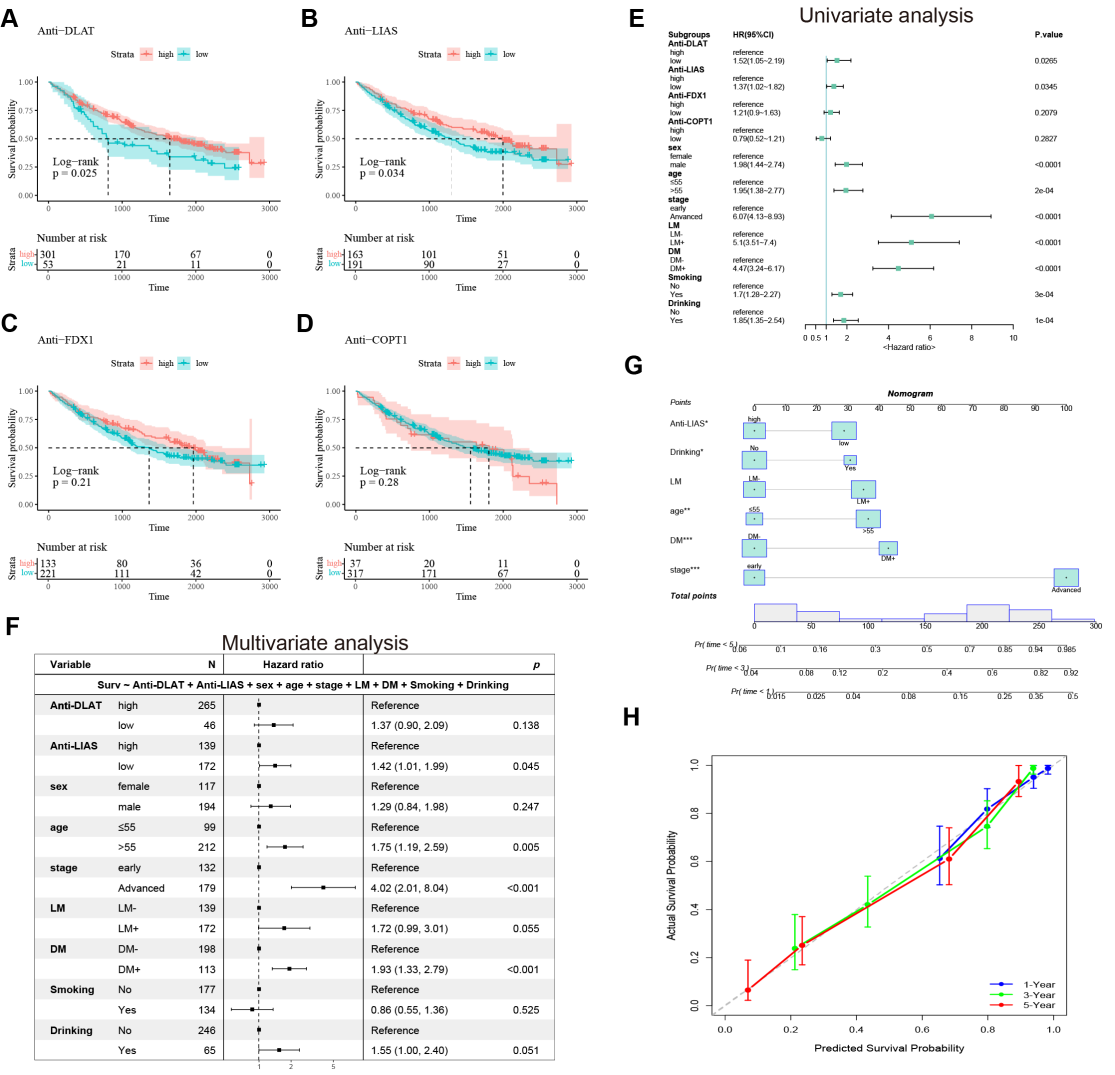
The impact of the autoantibody signature on the prognosis of NSCLC. (A–D) Survival curve of anti-DLAT, anti-LIAS, anti-FDX1, and anti-COPT1 autoantibodies. (E) Univariate analysis of the prognostic value of anti-DLAT and anti-LIAS autoantibodies. (F) Multivariate Cox regression analysis of the prognostic value of anti-DLAT and anti-LIAS autoantibodies. (G) Prognostic prediction model based on nomogram. (H) Calibration curve validation of the prognostic model. ^∗^, *P* < 0.05; ^∗∗^, *P* < 0.01; ^∗∗∗^, *P* < 0.001.

To investigate the impact of the autoantibody signature on the prognosis of NSCLC, we collected plasma samples from 354 NSCLC patients in the prognostic group and followed them up to determine their survival status (Table S1). Low expression of anti-DLAT and anti-LIAS autoantibodies significantly correlated with poor prognosis in patients with NSCLC (*P* < 0.05) (Figs. 6A–6B), whereas no significant difference was observed in the expression of anti-FDX1 and anti-COPT1 autoantibodies in relation to NSCLC prognosis (Figs. 6C–6D). These results suggest that anti-DLAT and anti-LIAS autoantibodies could serve as prognostic markers for NSCLC.

Univariate and multivariate Cox regression analyses provided a critical assessment of the impact of predictor variables on survival outcomes, informing prognostic evaluation and therapeutic strategies. Therefore, we evaluated the influence of various clinical factors (including anti-DLAT/LIAS autoantibodies, age, sex, tumor stage, and metastasis status) on the survival outcomes of NSCLC patients. Univariate Cox regression analysis revealed HRs of 1.52 (95% CI [1.05–2.19]) for anti-DLAT autoantibody and 1.37 (95% CI [1.02–1.82]) for anti-LIAS autoantibody (Fig. 6E). Subsequent multivariate analysis confirmed anti-LIAS autoantibody as an independent prognostic predictor (HR = 1.42, 95% CI [1.01–1.99]; Fig. 6F).

To further achieve individualized prognostic prediction, we constructed a nomogram model to predict the 1-year, 3-year, and 5-year survival rates (Fig. 6G). The calibration curves demonstrated good agreement between the predicted survival rates and the actual Kaplan–Meier survival rates at 1, 3, and 5 years (Fig. 6H). These findings confirmed that the anti-LIAS autoantibody is an independent biomarker for poor prognosis in NSCLC, and the integrated model demonstrated significant clinical utility for personalized outcome prediction.

## Discussion

The early diagnosis of NSCLC remains challenging owing to its asymptomatic presentation (Papavassiliou, Gogou & Papavassiliou, 2024; Relli et al., 2019). Autoantibody detection is a potential screening tool to increase the likelihood of early detection (Kim et al., 2024). This study systematically investigated the diagnostic and prognostic potential of autoantibodies targeting key cuproptosis-related proteins in NSCLC. Our results demonstrated significantly elevated levels of anti-DLAT and anti-LIAS autoantibodies in patients with NSCLC, indicating their robust diagnostic potential. To optimize diagnostic performance, we constructed a multi-autoantibody signature (anti-DLAT, anti-LIAS, anti-FDX1, and anti-COPT1 autoantibodies) to distinguish NSCLC from NC and BPN, achieving AUCs of 0.805 and 0.751, respectively. Finally, the correlation between the autoantibody signature and prognosis was validated in a prognostic cohort. Prognostic validation further established anti-LIAS autoantibody as an independent predictor of poor survival, and the integrated clinical nomogram demonstrated significant utility for personalized outcome prediction.

NSCLC is the most common type of lung cancer, characterized by high incidence and mortality rates (Leiter, Veluswamy & Wisnivesky, 2023; Patel et al., 2022). To address this challenge, the current focus of medicine has shifted toward early cancer screening and detection, enabling the implementation of radical treatment before cancer progresses to an advanced stage (Etzioni et al., 2003). Therefore, serum biomarkers that are detectable before cancer onset have attracted significant attention (Wagner, Verma & Srivastava, 2004), particularly those targeting specific TAAs (Anderson & LaBaer, 2005; Shiku et al., 1977). Autoantibodies hold significant value in the diagnosis and treatment of lung cancer (Liu, 2019). Liu et al. (2023) highlighted the clinical application value of a panel of seven autoantibodies (including p53, GAGE7, PGP9.5, CAGE, MAGEA1, SOX2 and GBU4-5) in promoting early lung cancer detection and prognostic evaluation. Additionally, autoantibodies against TAAs such as p53, NY-ESO-1, and SOX2, are significantly elevated in the serum of patients with lung cancer and correlate with tumor stage and prognosis (Chapman et al., 2008).

Copper is an essential enzymatic cofactor that is crucial for human physiological functions (Guo et al., 2025; Kahlson & Dixon, 2022; Parsanathan, 2024) and modulates tumor-associated signaling pathways (Li, 2020). Studies have shown that abnormal regulation of certain cuproptosis-related genes plays a significant role in the occurrence and development of cancer (Brändlein et al., 2003; Wu et al., 2021; Zhang et al., 2022). Dysfunction of DLAT and LIAS disrupts cellular copper homeostasis, promoting copper ion accumulation and inducing tumor cell death through impaired lipoylation-dependent metabolism (Chong et al., 2024; Dreishpoon et al., 2023; Huang et al., 2024). Abnormal DLAT expression has been reported in breast cancer and hepatocellular carcinomas (Liu et al., 2024a; Xu et al., 2025). In addition, aberrant LIAS expression has been observed in gastric cancer (Yan et al., 2023). Analyses based on the GEPIA and HPA databases revealed that DLAT and LIAS were highly expressed in NSCLC (Fig. S7). Collectively, these findings established that DLAT and LIAS are functional TAAs capable of eliciting elevated anti-DLAT and anti-LIAS autoantibodies responses in patients with NSCLC. In this study, we found that the anti-DLAT and anti-LIAS autoantibodies were highly expressed in NSCLC plasma, indicating their potential diagnostic utility for NSCLC detection.

However, single autoantibody detection exhibits inherent limitations in cancer screening, typically demonstrating sensitivities of only 10–30% against specific tumor-associated antigens (Casiano, Mediavilla-Varela & Tan, 2006; Tan et al., 2009). The combined detection of multiple autoantibodies and other biomarkers can improve the diagnostic accuracy of NSCLC (Ma et al., 2024; Tan et al., 2009). In this study, we constructed a multiplex autoantibody panel targeting DLAT, LIAS, FDX1, and COPT1, which significantly improved NSCLC discrimination from NC and BPN, with AUCs of 0.805 (95% CI [0.768–0.842]) and 0.751 (95% CI [0.710–0.793]), respectively. These results indicate that the multi-autoantibody signature significantly improves the diagnostic accuracy of NSCLC.

Smoking serves as a critical risk factor for lung cancer, particularly among individuals aged 50–80 years with a ≥ 20 pack-year smoking history. In the present study, we observed that both anti-DLAT and anti-LIAS autoantibodies demonstrated diagnostic potential for distinguishing NSCLC from BPN within this high-risk subgroup. Furthermore, the combined autoantibody signature achieved an AUC of 0.767 (95% CI [0.659–0.875], sensitivity = 60.8%, specificity = 85.7%) in distinguishing NSCLC from BPN. However, the sample size of this high-risk subgroup was relatively small, which may have affected the statistical power and increased the risk of incidental conclusions.

In addition to their diagnostic significance, autoantibodies can also be utilized for prognosis or monitoring of treatment responses, as many autoantibodies are associated with clinical outcomes (Hoshino et al., 2020). For instance, P53 autoantibodies may serve as significant predictive factors for lung cancer mortality (Ma et al., 2024). While these findings underscore the prognostic potential of autoantibodies in lung cancer, optimal clinical applications require integration with clinicopathological parameters to maximize predictive accuracy. The prognostic value of the autoantibody signature was investigated in a prospective cohort of 354 patients with NSCLC. The results revealed that diminished anti-DLAT and anti-LIAS autoantibodies levels were significantly correlated with a poor prognosis. The multivariable Cox regression model demonstrated that anti-LIAS autoantibody could serve as an independent prognostic factor for NSCLC, providing a basis for clinical decision-making and personalized treatment.

However, this study had certain limitations. The current findings remain preliminary as they were derived from pilot investigations with limited sample sizes and lack verification through multicenter trials. Although the specificity and sensitivity of this study demonstrated some capability to distinguish NSCLC from other groups, these values suggest the possibility of false-negative and false-positive results. The primary clinical value of a biomarker intended to complement low-dose computed tomography (LDCT) screening is its ability to refine the characterization of early-stage ambiguous cases. Future efforts will prioritize optimizing the model’s validation in populations with genuine screening uncertainty, such as patients with LDCT-detected indeterminate nodules that do not yet meet the established high-risk criteria. The lack of smoking history data in the NC group limited the ability to perform stratified analyses according to smoking status, potentially limiting the generalizability of our findings. Moving forward, we plan to conduct prospective multicenter cohort studies with statistical completeness and expanded sample sizes to systematically validate the diagnostic robustness and clinical applicability of our model.

In conclusion, we demonstrated for the first time the diagnostic efficacy of anti-DLAT and anti-LIAS autoantibodies for NSCLC detection. Anti-DLAT and anti-LIAS autoantibodies were highly expressed in NSCLC and could distinguish NSCLC from NC and BPN. To optimize the diagnostic efficacy, we integrated anti-FDX1 and anti-COPT1 autoantibodies to construct a novel autoantibody signature that significantly improved the diagnostic efficacy for NSCLC. Finally, prognostic validation further established that the anti-LIAS autoantibody could serve as an independent prognostic factor for NSCLC, providing a basis for clinical decision-making and personalized treatment. Collectively, these findings suggest that the autoantibody signature targeting cuproptosis-related proteins has the potential for NSCLC diagnosis and precise prognostic assessment.

## Supplemental Information

10.7717/peerj.21260/supp-1 Supplemental Information 1 Raw data Sheet 1: Raw data of the NC verification group; Sheet 2: Raw data of the NSCLC verification group; Sheet 3: Raw data of the NC validation group; Sheet 4: Raw data of the BPN validation group; Sheet 5: Raw data of the NSCLC validation group; Sheet 6: Raw data for prognosis. NC, normal control; BPN, patients with benign pulmonary nodule; NSCLC, non-small cell lung cancer.

10.7717/peerj.21260/supp-2 Supplemental Information 2 Clinical characteristics of the prognostic group NSCLC, non-small cell lung cancer.

10.7717/peerj.21260/supp-3 Supplemental Information 3 The diagnostic efficacy of the autoantibody signature in NSCLC *vs* NC and BPN. Se, sensitivity; Sp, specificity; AR, agreement rate; CI, confidence interval

10.7717/peerj.21260/supp-4 Supplemental Information 4 The diagnostic efficacy of the autoantibody signature in NSCLC *vs* NC and BPN Se, sensitivity; Sp, specificity; AR, agreement rate; CI, confidence interval; Smoking ≥ 20, individuals with smoking history ≥ 20 pack-year.

10.7717/peerj.21260/supp-5 Supplemental Information 5 The overall study design NSCLC, non-small cell lung cancer; NC, normal control; BPN, benign pulmonary nodule; ELISA, enzyme-linked immunosorbent assay.

10.7717/peerj.21260/supp-6 Supplemental Information 6 The expression levels of anti-DLAT and anti-LIAS autoantibodies in different clinical subgroups LM, lymph node metastasis; DM, distant metastasis; LUAD, lung adenocarcinoma; LUSC, lung squamous cell carcinoma.

10.7717/peerj.21260/supp-7 Supplemental Information 7 The diagnostic efficacy of anti-DLAT and anti-LIAS autoantibodies in different clinical subgroups and NC Clinical subgroups including: Anti-DLAT: Early (A), Advanced (B), LM + (C), LM- (D), DM+ (E), DM- (F), LUSC (G), LUAD (H). Anti-LIAS: Early (I), Advanced (J), LM+ (K), LM- (L), DM+ (M), DM- (N), LUSC (O), LUAD (P). LM, lymph node metastasis; DM, distant metastasis; LUAD, lung adenocarcinoma; LUSC, lung squamous cell carcinoma.

10.7717/peerj.21260/supp-8 Supplemental Information 8 The diagnostic efficacy of anti-DLAT and anti-LIAS autoantibodies stratified by age, gender Anti-DLAT: Male (A) and Female (B); anti-LIAS: Male (C) and Female (D); anti-DLAT: Age < 50 years old (E) and 50 ≤ Age ≤ 80 years old (F); anti-LIAS: Age < 50 years old (G) and 50 ≤ Age ≤ 80 years old (H). Se, sensitivity; Sp, specificity.

10.7717/peerj.21260/supp-9 Supplemental Information 9 The diagnostic efficacy of anti-DLAT and anti-LIAS autoantibodies in different clinical subgroups and BPN Clinical subgroups including: Anti-DLAT: Early (A), Advanced (B), LM+ (C), LM- (D), DM+ (E), DM- (F), LUSC (G), LUAD (H). Anti-LIAS: Early (I), Advanced (J), LM+ (K), LM- (L), DM+ (M), DM- (N), LUSC (O), LUAD (P). LM, lymph node metastasis; DM, distant metastasis; LUAD, lung adenocarcinoma; LUSC, lung squamous cell carcinoma.

10.7717/peerj.21260/supp-10 Supplemental Information 10 The diagnostic efficacy of anti-DLAT and anti-LIAS autoantibodies stratified by age, gender, smoking and drinking status Anti-DLAT: Male (A) and Female (B); anti-LIAS: Male (C) and Female (D); anti-DLAT: Age < 50 years old (E) and 50 ≤ Age ≤ 80 years old (F); anti-LIAS: Age < 50 years old (G) and 50 ≤ Age ≤ 80 years old (H). Anti-DLAT: Smoking-yes (I), Smoking-no (J), Drinking-yes (K), Drinkig-no (L); anti-LIAS: Smoking-yes (M), Smoking-no (N), Drinking-yes (O), Drinkig-no (P). Se, sensitivity; Sp, specificity; Smoking yes/no, individuals with or without smoking history; Drinking yes/no, individuals with or without drinking history.

10.7717/peerj.21260/supp-11 Supplemental Information 11 DLAT and LIAS was highly expressed in patients with NSCLC (A) IHC staining images of DLAT from HPA database in normal tissues and NSCLC tissues. (B) IHC staining images of DLAT from HPA database in normal tissues and NSCLC tissues. (C) The expression of * DLAT* in normal tissues, LUAD and LUSC from TCGA and GTEx databases. (D) The expression of * LIAS* in normal tissues, LUAD and LUSC from TCGA and GTEx databases. HPA, The Human Protein Atlas; LUAD, lung adenocarcinoma; LUSC, lung squamous cell carcinoma; T, tumor; N, normal.

## Additional Information and Declarations

### Data Availability

The following information was supplied regarding data availability: The raw data are available in the Supplementary File.
