# Supplementary material for: An autoantibody signature targeting cuproptosis-related proteins for non-small cell lung cancer detection and prognosis
Source: PeerJ. 2026 May 27;14:e21260. doi: 10.7717/peerj.21260 (PMC13221990; doi:10.7717/peerj.21260)
Supplement: Supplemental Information 2 — NSCLC, non-small cell lung cancer. [file peerj-14-21260-s002.docx]

**Supplementary Table 1. Clinical characteristics of the prognostic group.**

| **NSCLC（N=354)** | | | |
| --- | --- | --- | --- |
| **Age (year), n (%)** | | **Gender, n (%)** | |
| ≤ 55y | 108 (30.51) | Male | 225 (63.56) |
| > 55y | 246 (69.49) | Female | 129 (36.44) |
| **Clinical stage, n (%)** | | **Histology, n (%)** | |
| Early | 147 (41.53) | LUAD | 260 (73.45) |
| Advanced | 192 (54.24) | LUSC | 92 (25.99) |
| Unknown | 15 (4.23) | Other NSCLC | 2 (0.56） |
| **Smoking, n (%)** | | **Drinking, n (%)** | |
| Yes | 155 (43.79) | Yes | 74 (20.90) |
| No | 194 (54.80) | No | 273 (77.12) |
| Unknown | 5 (1.41) | Unknown | 7 (1.98) |
| **Lymph node metastasis, n (%)** | | **Distant metastasis, n (%)** | |
| Yes | 184 (51.98) | Yes | 116 (32.77) |
| No | 150 (42.37) | No | 204 (57.63) |
| Unknown | 20 (8.47) | Unknown | 34 (9.60) |
| **Survival status, n (%)** | |  | |
| Alive | 165 (46.60) |  | |
| Dead | 189 (53.30) |  | |

NSCLC, non-small cell lung cancer.
