# Supplementary material for: An autoantibody signature targeting cuproptosis-related proteins for non-small cell lung cancer detection and prognosis
Source: PeerJ. 2026 May 27;14:e21260. doi: 10.7717/peerj.21260 (PMC13221990; doi:10.7717/peerj.21260)
Supplement: Supplemental Information 3 [file peerj-14-21260-s003.docx]

**Supplementary Table 2. Diagnostic efficacy of the autoantibody signature in NSCLC vs NC and BPN.**

|  |  | AUC | 95% CI | Se (%) | Sp (%) | AR (%) |
| --- | --- | --- | --- | --- | --- | --- |
| NSCLC vs NC | Anti-DLAT | 0.712 | 0.669-0.756 | 49.6 | 80.0 | 64.6 |
|  | Anti-LIAS | 0.668 | 0.623-0.714 | 94.6 | 33.5 | 63.9 |
|  | Anti-FDX1 | 0.636 | 0.589-0.683 | 88.5 | 33.5 | 61.0 |
|  | Anti-COPT1 | 0.636 | 0.588-0.683 | 35.0 | 85.4 | 60.0 |
|  | Anti-DLAT/LIAS/FDX1/COPT1 | 0.805 | 0.768-0.842 | 67.3 | 79.2 | 73.1 |
|  |  |  |  |  |  |  |
| NSCLC vs BPN | Anti-DLAT | 0.695 | 0.650-0.739 | 79.2 | 50.0 | 64.6 |
|  | Anti-LIAS | 0.636 | 0.588-0.683 | 90.8 | 30.4 | 60.6 |
|  | Anti-FDX1 | 0.553 | 0.503-0.602 | 88.1 | 27.7 | 57.9 |
|  | Anti-COPT1 | 0.641 | 0.594-0.688 | 69.2 | 52.7 | 61.0 |
|  | Anti-DLAT/LIAS/FDX1/COPT1 | 0.751 | 0.710-0.793 | 61.2 | 78.1 | 69.4 |

Se, sensitivity; Sp, specificity; AR, agreement rate; CI, confidence interval.
