# Supplementary material for: An autoantibody signature targeting cuproptosis-related proteins for non-small cell lung cancer detection and prognosis
Source: PeerJ. 2026 May 27;14:e21260. doi: 10.7717/peerj.21260 (PMC13221990; doi:10.7717/peerj.21260)
Supplement: Supplemental Information 4 — Se, sensitivity; Sp, specificity; AR, agreement rate; CI, confidence interval; Smoking ≥ 20, individuals with smoking history ≥ 20 pack-year. [file peerj-14-21260-s004.docx]

**Supplementary Table 3. The diagnostic efficacy of the autoantibody signature in NSCLC vs NC and BPN.**

|  |  | AUC | 95% CI | Se | Sp | AR |
| --- | --- | --- | --- | --- | --- | --- |
|  |  |  |  | (%) | (%) | (%) |
| NSCLC (Early) vs NC | Anti-DLAT | 0.709 | 0.659-0.760 | 46.8 | 83.1 | 69.1 |
|  | Anti-LIAS | 0.672 | 0.621-0.723 | 94.9 | 33.5 | 56.5 |
|  | Anti-FDX1 | 0.623 | 0.569-0.678 | 87.3 | 33.5 | 53.8 |
|  | Anti-COPT1 | 0.615 | 0.560-0.672 | 31.6 | 86.2 | 65.1 |
|  | Anti-DLAT/LIAS/FDX1/COPT1 | 0.797 | 0.753-0.842 | 65.2 | 80.8 | 74.6 |
|  |  |  |  |  |  |  |
| NSCLC (Early) vs BPN | Anti-DLAT | 0.692 | 0.641-0.744 | 78.5 | 50.0 | 60.8 |
|  | Anti-LIAS | 0.639 | 0.586-0.692 | 90.8 | 30.4 | 55.5 |
|  | Anti-FDX1 | 0.541 | 0.486-0.597 | 86.7 | 27.7 | 50.0 |
|  | Anti-COPT1 | 0.622 | 0.568-0.677 | 67.1 | 52.7 | 58.1 |
|  | Anti-DLAT/LIAS/FDX1/COPT1 | 0.744 | 0.695-0.793 | 81.0 | 56.9 | 65.8 |
|  |  |  |  |  |  |  |
| NSCLC vs BPN  (Age:50-80 years; Smoking ≥ 20) | Anti-DLAT | 0.673 | 0.544-0.802 | 86.3 | 50.0 | 70.89 |
|  | Anti-LIAS | 0.729 | 0.613-0.845 | 58.8 | 82.1 | 69.62 |
|  | Anti-FDX1 | 0.657 | 0.526-0.788 | 82.4 | 50.0 | 70.89 |
|  | Anti-COPT1 | 0.659 | 0.530-0.788 | 70.6 | 57.1 | 67.09 |
|  | Anti-DLAT/LIAS/FDX1/COPT1 | 0.767 | 0.659-0.875 | 60.8 | 85.7 | 70.89 |

Se, sensitivity; Sp, specificity; AR, agreement rate; CI, confidence interval; Smoking ≥ 20, individuals with smoking history ≥ 20 pack-year.
