# Supplementary figures and images for: An autoantibody signature targeting cuproptosis-related proteins for non-small cell lung cancer detection and prognosis
Source: PeerJ. 2026 May 27;14:e21260. doi: 10.7717/peerj.21260 (PMC13221990; doi:10.7717/peerj.21260)

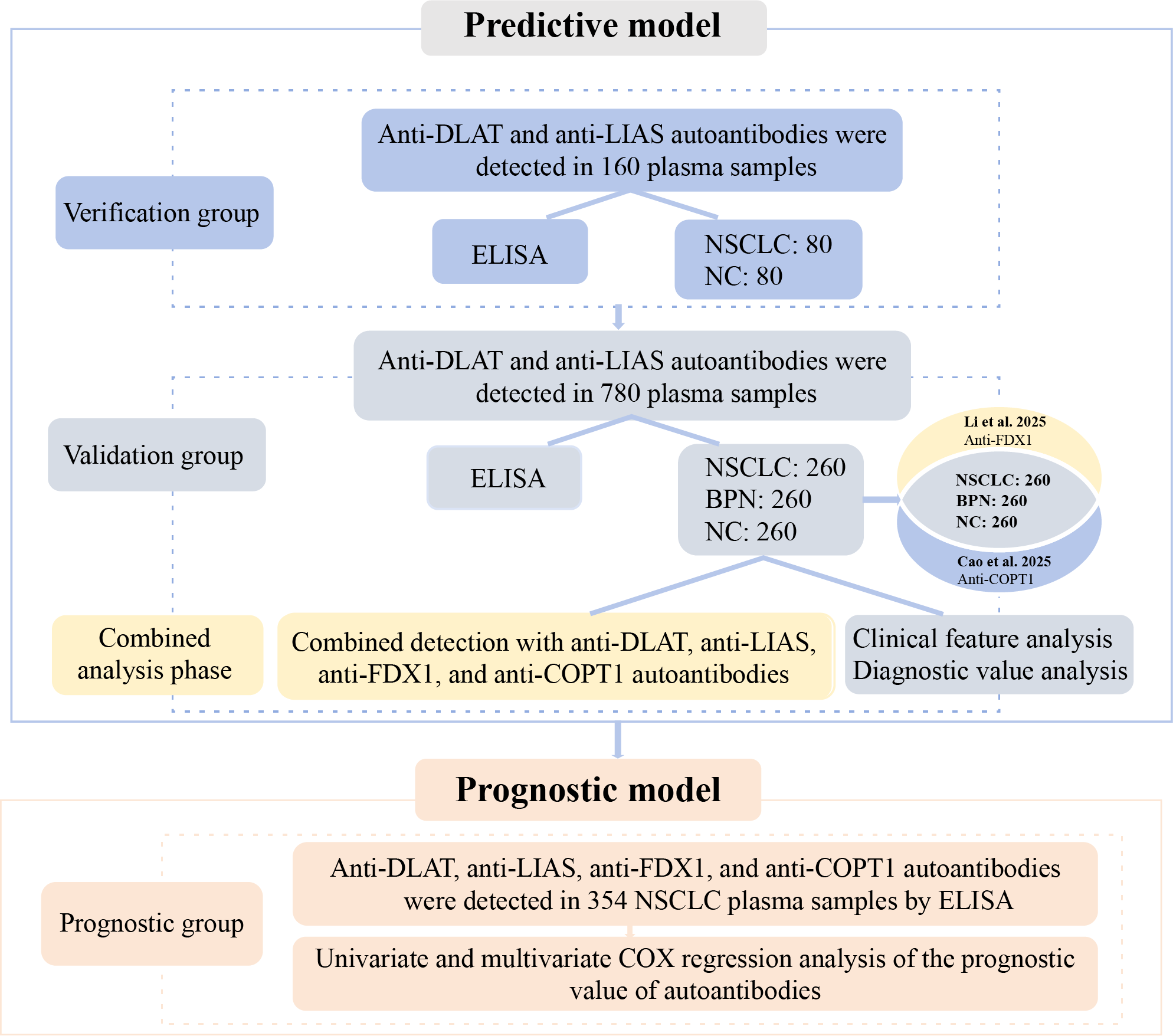

Supplement: Supplemental Information 5 — NSCLC, non-small cell lung cancer; NC, normal control; BPN, benign pulmonary nodule; ELISA, enzyme-linked immunosorbent assay. [file peerj-14-21260-s005.png]

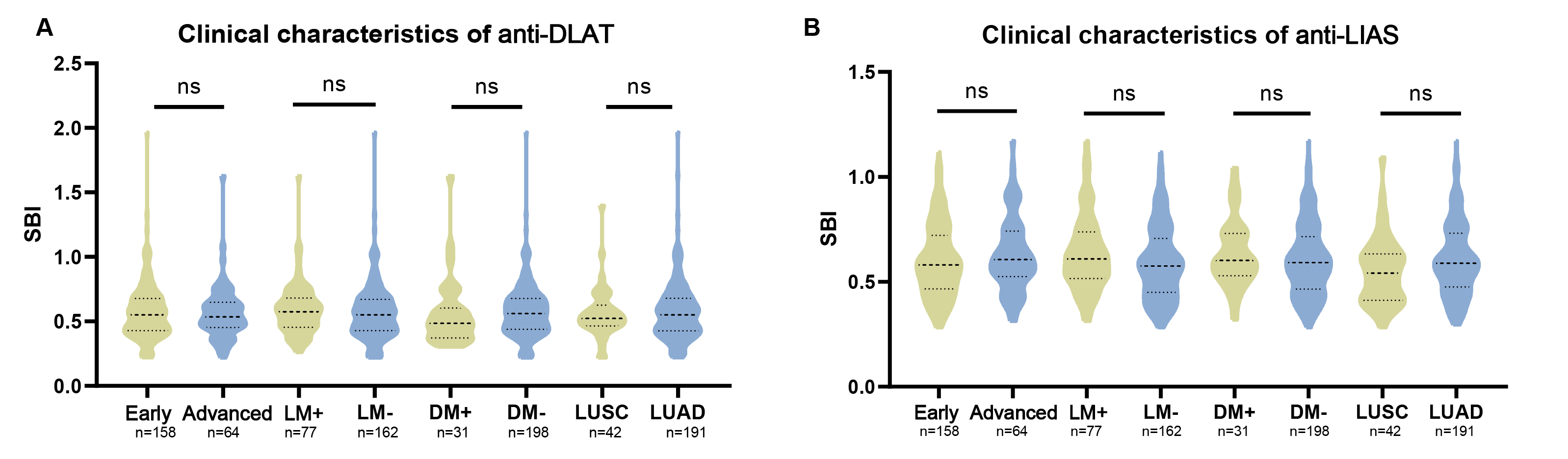

Supplement: Supplemental Information 6 — LM, lymph node metastasis; DM, distant metastasis; LUAD, lung adenocarcinoma; LUSC, lung squamous cell carcinoma. [file peerj-14-21260-s006.png]

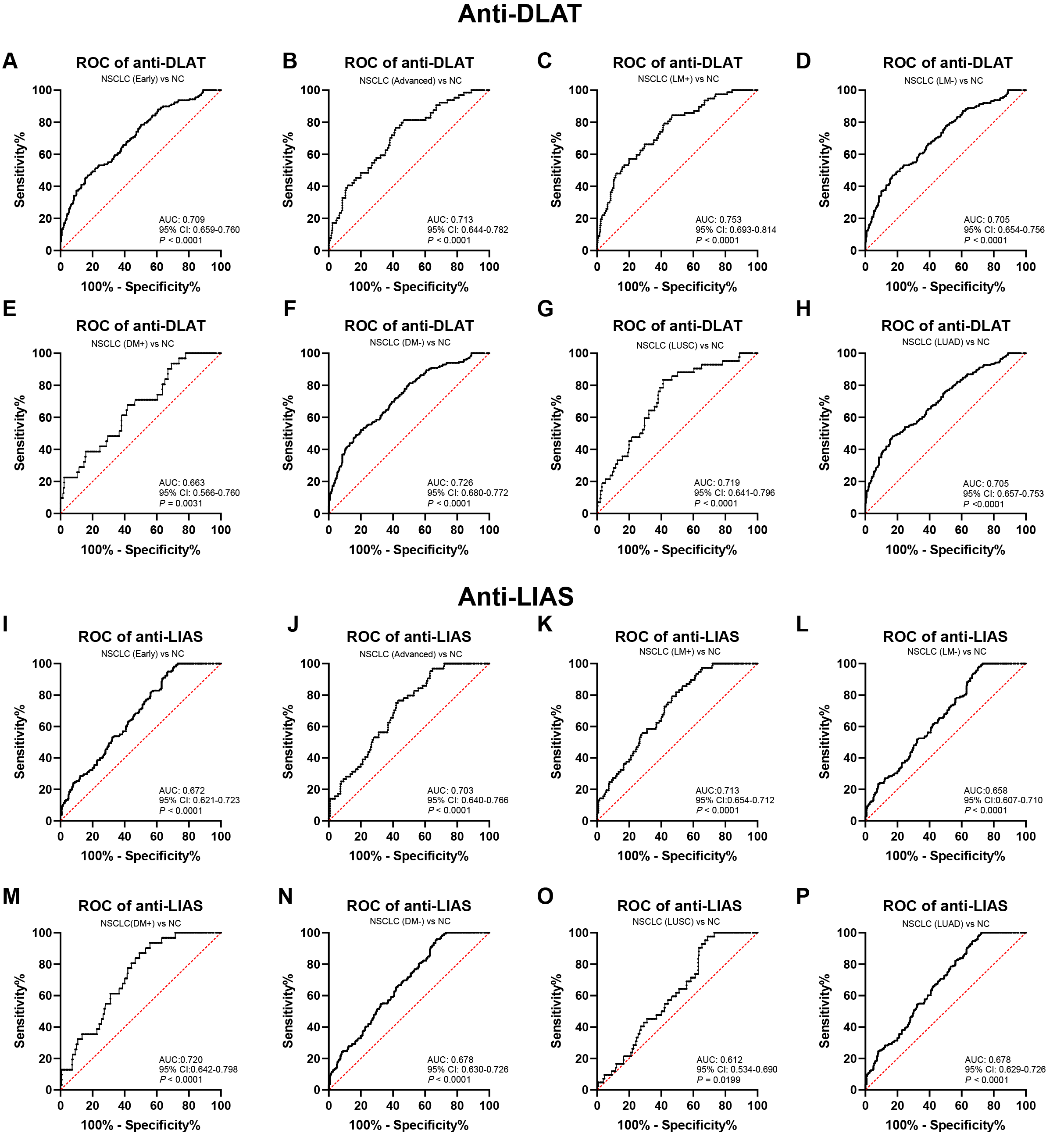

Supplement: Supplemental Information 7 — Clinical subgroups including: Anti-DLAT: Early (A), Advanced (B), LM + (C), LM- (D), DM+ (E), DM- (F), LUSC (G), LUAD (H). Anti-LIAS: Early (I), Advanced (J), LM+ (K), LM- (L), DM+ (M), DM- (N), LUSC (O), LUAD (P). LM, lymph node metastasis; DM, distant metastasis; LUAD, lung adenocarcinoma; LUSC, lung squamous cell carcinoma. [file peerj-14-21260-s007.png]

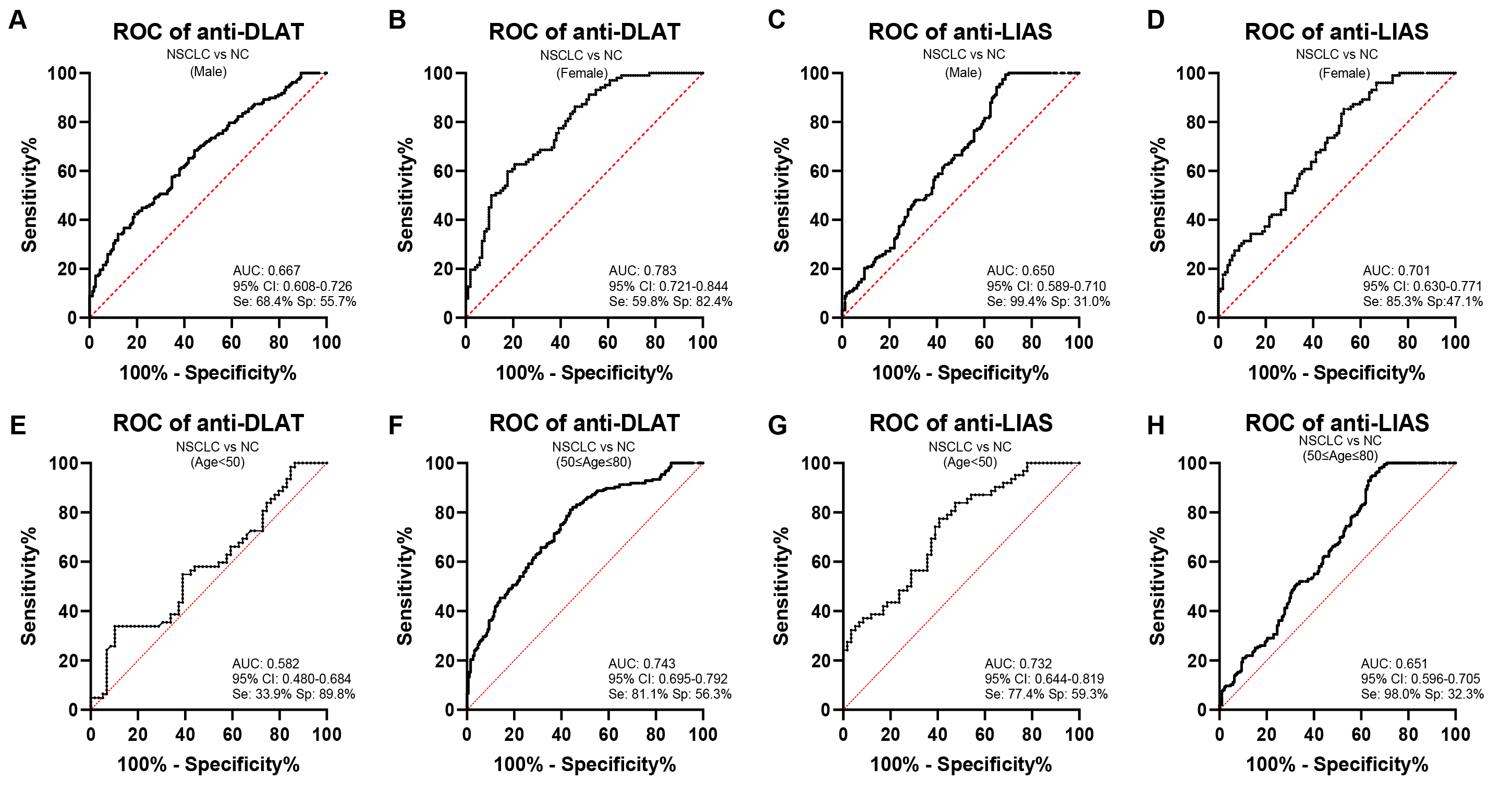

Supplement: Supplemental Information 8 — Anti-DLAT: Male (A) and Female (B); anti-LIAS: Male (C) and Female (D); anti-DLAT: Age < 50 years old (E) and 50 ≤ Age ≤ 80 years old (F); anti-LIAS: Age < 50 years old (G) and 50 ≤ Age ≤ 80 years old (H). Se, sensitivity; Sp, specificity. [file peerj-14-21260-s008.png]

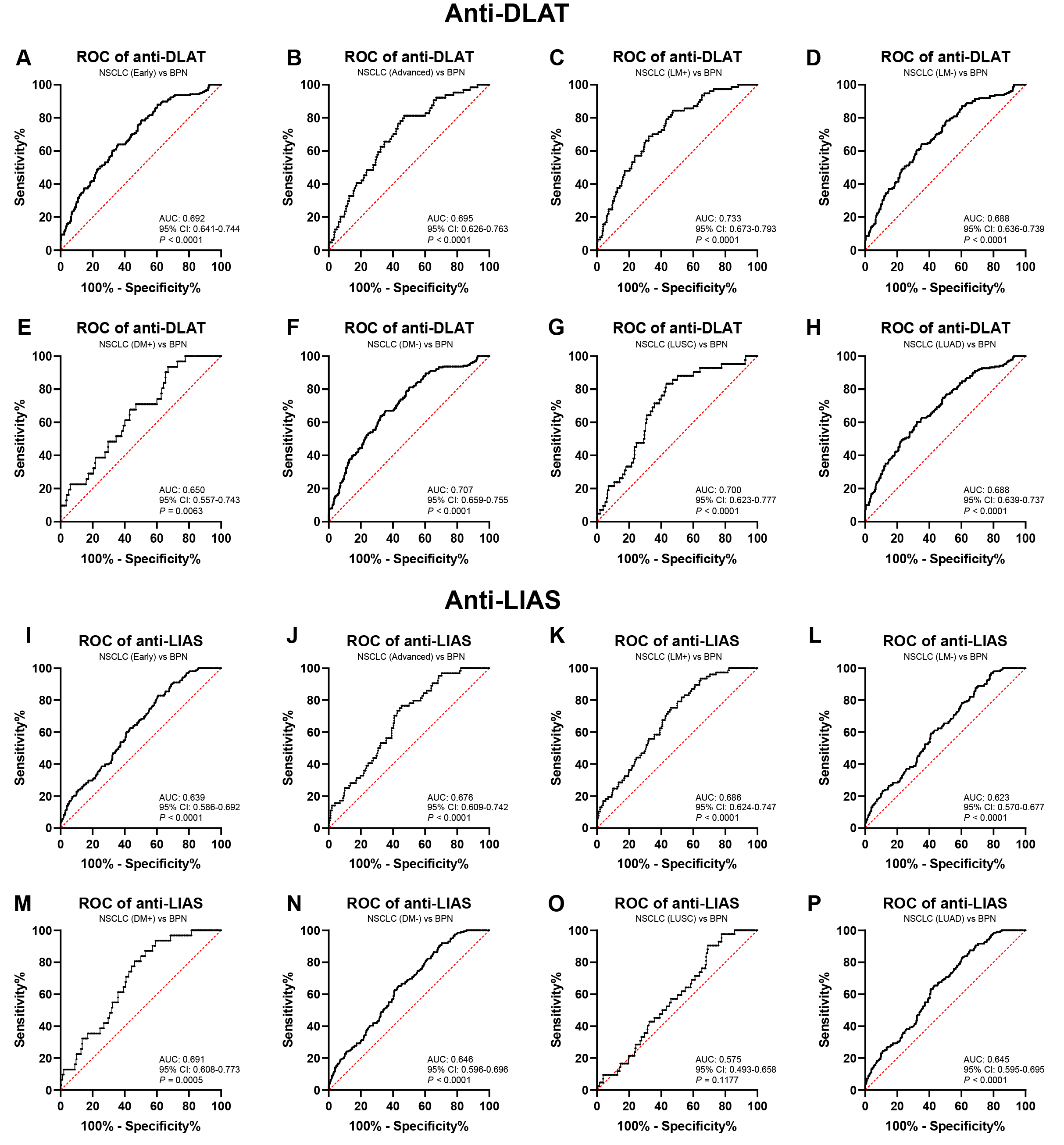

Supplement: Supplemental Information 9 — Clinical subgroups including: Anti-DLAT: Early (A), Advanced (B), LM+ (C), LM- (D), DM+ (E), DM- (F), LUSC (G), LUAD (H). Anti-LIAS: Early (I), Advanced (J), LM+ (K), LM- (L), DM+ (M), DM- (N), LUSC (O), LUAD (P). LM, lymph node metastasis; DM, distant metastasis; LUAD, lung adenocarcinoma; LUSC, lung squamous cell carcinoma. [file peerj-14-21260-s009.png]

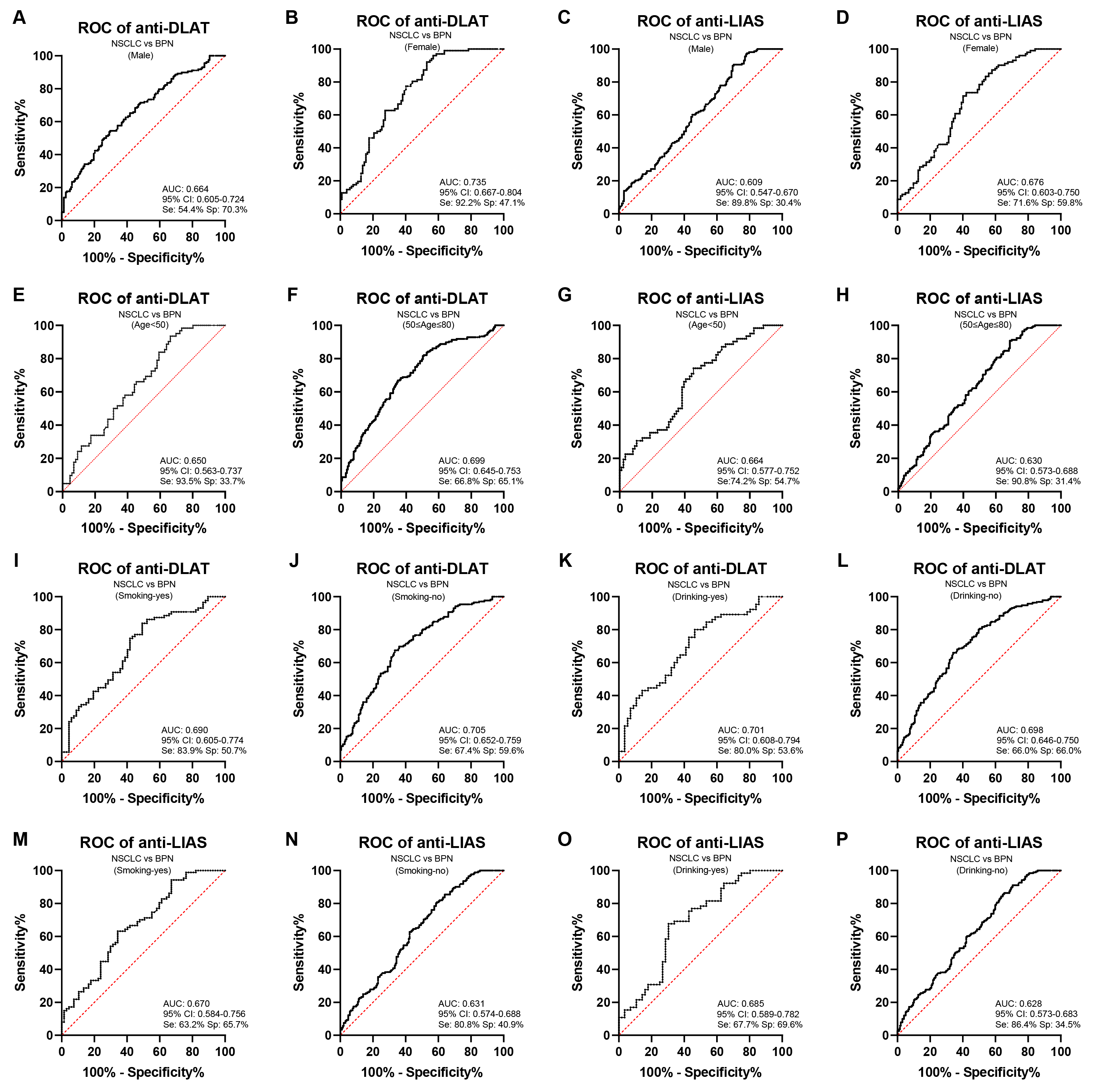

Supplement: Supplemental Information 10 — Anti-DLAT: Male (A) and Female (B); anti-LIAS: Male (C) and Female (D); anti-DLAT: Age < 50 years old (E) and 50 ≤ Age ≤ 80 years old (F); anti-LIAS: Age < 50 years old (G) and 50 ≤ Age ≤ 80 years old (H). Anti-DLAT: Smoking-yes (I), Smoking-no (J), Drinking-yes (K), Drinkig-no (L); anti-LIAS: Smoking-yes (M), Smoking-no (N), Drinking-yes (O), Drinkig-no (P). Se, sensitivity; Sp, specificity; Smoking yes/no, individuals with or without smoking history; Drinking yes/no, individuals with or without drinking history. [file peerj-14-21260-s010.png]

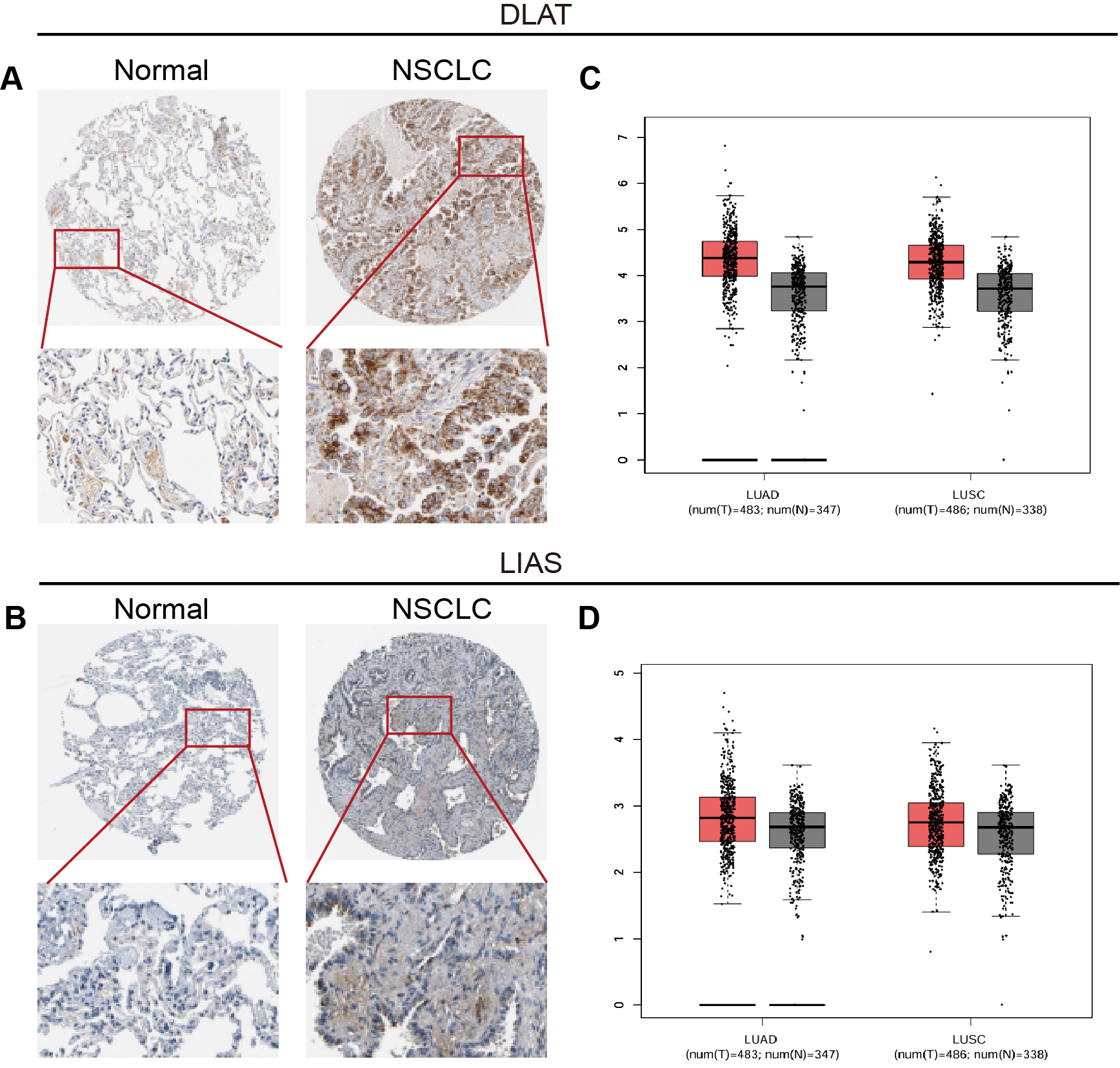

Supplement: Supplemental Information 11 — (A) IHC staining images of DLAT from HPA database in normal tissues and NSCLC tissues. (B) IHC staining images of DLAT from HPA database in normal tissues and NSCLC tissues. (C) The expression of DLAT in normal tissues, LUAD and LUSC from TCGA and GTEx databases. (D) The expression of LIAS in normal tissues, LUAD and LUSC from TCGA and GTEx databases. HPA, The Human Protein Atlas; LUAD, lung adenocarcinoma; LUSC, lung squamous cell carcinoma; T, tumor; N, normal. [file peerj-14-21260-s011.png]
